# The Role of Specific Checkpoint-Induced S-Phase Transcripts in Resistance to Replicative Stress

**DOI:** 10.1371/journal.pone.0006944

**Published:** 2009-09-11

**Authors:** Chaitali Dutta, Nicholas Rhind

**Affiliations:** Department of Biochemistry and Molecular Pharmacology, University of Massachusetts Medical School, Worcester, Massachusetts, United States of America; University College London, United Kingdom

## Abstract

Checkpoint activation during S phase modulates transcription. In response to replication arrest, the fission yeast Cds1 checkpoint kinase maintains the normal S-phase transcriptional program by regulating MBF, the S-phase transcription factor. We show that similar regulation occurs in response to DNA damage during S-phase. We test the relative contributions to replication-stress resistance of transcriptional regulation and the two other major checkpoint functions: cell-cycle arrest and fork stabilization. We show that, although transcriptional regulation provides only modest resistance relative to fork stabilization, it contributes significantly to cell survival. Finally, we investigate the roles of two specific transcripts: *mik1* and *mrc1*. These results demonstrate the general importance of checkpoint regulation of G1/S transcription in response to replicative stress and elucidate the specific roles of Mik1 and Mrc1 in the checkpoint.

## Introduction

In response to inhibition of DNA replication, the replication checkpoint arrests the cell cycle before mitosis, stabilizes stalled replication forks and regulates S-phase transcription [1], [2]. The transcriptional branch of the checkpoint response up-regulates genes thought to be important for cells to survive prolonged replication arrest. The replication checkpoint, also known as the S-M checkpoint, is commonly induced by hydroxyurea (HU) treatment. Hydroxyurea is a competitive inhibitor of ribonucleotide reductase which interferes with deoxyribonucleotide synthesis, resulting in deoxyribonucleotide depletion, fork stalling and checkpoint activation [3]. A related checkpoint is activated by DNA damage during replication. The S-phase DNA damage checkpoint, also known as the intra-S checkpoint, is commonly induced by methyl methanesulfonate (MMS) treatment, which methylates DNA, generating adducts that are recognized as damage primarily during S phase.

The replication checkpoint and S-phase DNA damage checkpoints are mechanistically closely related. Whether they are two distinct checkpoints or one checkpoint activated by different replication stresses is somewhat of a semantic question. They both rely on the central checkpoint kinase Rad3, the fission yeast homolog of human ATR and budding yeast Mec1, and the downstream checkpoint effector kinase Cds1, homolog of Chk2 and Rad53. They have the same two major functions: cell-cycle arrest and replication fork stabilization. However, it is unclear if the mechanisms of fork stabilization are the same. Furthermore, the replication checkpoint regulates transcription by maintaining the normal G1/S transcriptional program through regulation of MBF, the S-phase transcription factor [4], [5]. This function has been reported in response to S-phase DNA damage that block replication forks [4], but not for general DNA damage, such a IR-induced double-stand breaks, which are thought to activate the checkpoint independently of replication forks.

Although checkpoint regulation of transcription has been shown to contribute to resistance to replication stress [4], [5], it is not clear how important it is relative to the other checkpoint functions. Furthermore, it is unclear which of the approximately 20 MBF-dependent G1/S transcripts involved in origin licensing, replication, repair and other functions are important for the checkpoint function or what role they might play in the checkpoint [5]–[7]. We address these questions by showing that the S-phase DNA damage checkpoint regulates S-phase transcription, by directly testing the relative importance of cell-cycle arrest, fork stability and transcriptional regulation and by identifying the roles of specific transcripts in the checkpoint.

## Materials and Methods

### Cell culture

The strains used are listed in Table 1. They were created and maintained using standard methods and conditions [8]. Briefly, cells were grown in yeast extract with supplements (YES) media at 30°C, with the exception of yFS632 cells, which were grown in Edinburgh minimal media supplemented with leucine, uracil, adenine and histidine (LUAH) and with thiamine as needed. The expression from the *nmt1* promoter was induced by growing cells in LUAH without thiamine for 16–18 hours at 30°C. For time courses, cells were synchronized by centrifugal elutriation and treated with 10 mM hydroxyurea or 200 grays ionizing radiation (IR) at time 0 or 0.015% MMS at 60 minutes. For IR, a Faxitron Cabinet X-ray system Model RX-650 was used at 10 Gray/minute. The delay in MMS treatment prevents cells form arresting in the first G2. For acute sensitivity assays, cells were incubated with either 10 mM HU or 0.03% MMS for the indicated times before plating. Colonies were counted after 7 days at 30°C.

**Table 1:** List of strains.

yFS625	*h- leu1-32 ura4-D18 cdc10 (kanMX6)*
yFS626	*h+ leu1-32 ura4-D18 cdc10-2E (kanMX6)*
yFS627	*h- leu1-32 ura4-D18 cdc10 (kanMX6) cds1::ura4*
yFS628	*h- leu1-32 ura4-D18 cdc10 (kanMX6) rad3::ura4*
yFS629	*h- leu1-32 ura4-D18 cdc10-2E (kanMX6) cds1::ura4*
yFS630	*h+ leu1-32 ura4-D18 cdc10-2E (kanMX6) rad3::ura4*
yFS632	*h+ leu1-32 ura4-D18 mik1::ura4 nmt1:pyp3 (kanMX6)*
yFS643	*h+ leu1-32 ura4-D18 ade6-704 cdc10-2E (kanMX6) rad3::ura4 mik1::LEU2*
yFS644	*h+ leu1-32 ura4-D18 cdc10-2E (kanMX6) cds1::ura4 mik1::LEU2*
yFS645	*h+ leu1-32 ura4-D18 cdc10-2E (kanMX6) rad3::ura4 mrc1:kanMX6*
yFS646	*h+ leu1-32 ura4-D18 cdc10-2E (kanMX6) cds1::ura4 mrc1:ura4*

### RNA analysis

RNA was prepared for Northern blots probed with random-prime labeled *cdc22*, stripped and reprobed with *adh1* [5]. *cdc22* levels were normalized to *adh1* and then all time courses were normalized to asynchronous wild-type controls included on all gels. The 20 minute time point for the wild-time course was set to 1.

### Flow cytometry

Asynchronous cells were grown to an OD_600_ 1.0, sampled before adding 10 mM HU, then collected after 4 hours of HU treatment. Cells were washed once, resuspended in fresh media, and sampled every 20 minutes. Collected cells were fixed in 70% ethanol and processed for flow cytometry. Isolated nuclei were prepared as previously described and analyzed on a Becton-Dickinson FACScan flow cytometer [9]. The average S-phase progression was calculated by measuring the mean of the S-phase peak as a percentage of the position of between the means of the 1C and 2C contents.

## Results

### *cdc22* Transcription is Maintained During Activation of the S-Phase DNA Damage Checkpoint but not by Activation of the G2 DNA Damage Checkpoint

**Figure 1:**
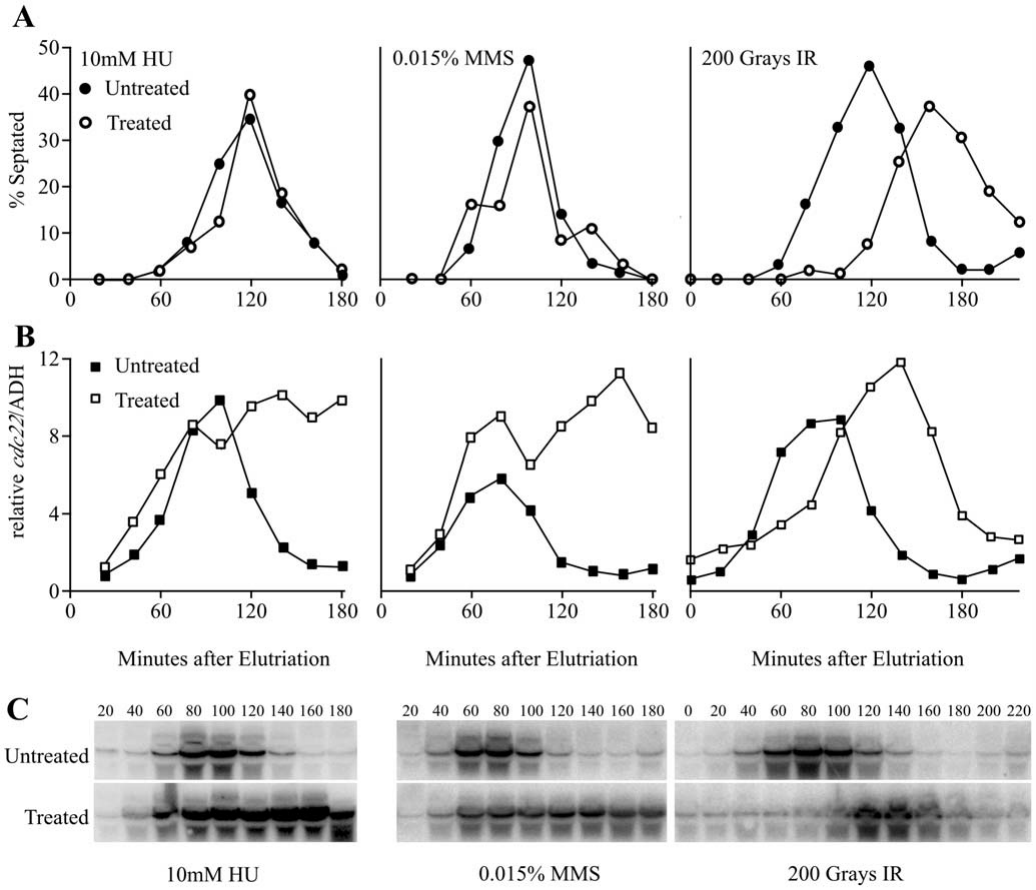
*cdc22* mRNA is up regulated during an HU-induced replication checkpoint and an MMS-induced S-phase DNA damage checkpoint, but not during an IR-induced G2 DNA damage checkpoint. Wild type (yFS625) cells were synchronized in early G2 by centrifugal elutriation, treated with 10 mM HU, 0.015% MMS or 200 grays of ionizing radiation and followed through a synchronous cell cycle. Samples were taken every 20 minutes for RNA isolation and visual inspection of septation. A) Septation index of treated cultures and untreated controls. B) *cdc22* mRNA level in treated cultures and untreated controls, normalized to *adh1* levels and to the 20 minute timepoint of the untreated sample. C) Northern blots quantitated in B. Blots were probed with *cdc22*, stripped and reprobed with *adh1* as a loading control.

Blocking replication by HU treatment up-regulates G1/S gene transcription [4]–[7], [10]. To determine if other S-phase checkpoints induce a transcriptional response, we followed the expression of *cdc22*, an MBF-dependent transcript encoding ribonucleotide reductase. RNA from cells synchronized in G2 by elutriation and treated with 10 mM HU or 0.015% MMS was analyzed by Northern blotting. As shown in Figure 1, both HU and MMS treated cells maintained *cdc22* mRNA level when arrested in S phase. However, neither checkpoint induced G1/S transcription in G2 prior to passage through mitosis and entry into the subsequent S phase.

In order to determine if checkpoint-induced transcription is also activated by the G2 DNA damage checkpoint, we irradiated G2 synchronized cells. We followed septation index and *cdc22* transcript levels every 20 minutes after elutriation (Figure 1). Cells treated with 200 Gray of ionizing radiation septated 40 minutes later than untreated cells (120 minutes and 160 minutes in control and treated sample, respectively). We observed a similar G1/S peak of *cdc22* expression 40 minutes later in irradiated cells than untreated cells (100 minutes and 140 minutes control and treated, respectively). However, we failed to observe any measurable increase of *cdc22* transcripts during G2. Therefore, our data suggest that MBF-dependent transcription is activated by the checkpoint only during S phase and not during G2.

### Checkpoint Dependent Regulation of G1/S Phase Transcription is Beneficial during Replication Stress

Activation of the S-phase checkpoints lead to three major outcomes: a cell-cycle arrest before mitosis, stabilization of replication forks and maintenance of G1/S transcription. To investigate the importance of checkpoint-mediated transcription relative to the other two functions, we built strains that lack one or more functions but maintained the other functions of the checkpoints (Table 2). For a strain that lacks all three functions of the checkpoints, we used *rad3*Δ, which lacks the central checkpoint kinase. For a strain that is compromised for the transcriptional response and fork stabilization function but proficient in preventing mitosis, we used *cds1*Δ; this stain lacks the S-phase checkpoints but is able to arrest in G2 via the Chk1-dependent G2 DNA damage checkpoint [11]. For a strain that lacks the cell-cycle arrest and fork stabilization functions, but has constitutive S-phase transcription that mimics checkpoint-activated MBF-dependent transcription, we used *rad3*Δ *cdc10-2E*. *cdc10-2E* encodes a version of the Cdc10 subunit of the MBF G1/S transcription factor containing two serine-to-glutamic-acid substitutions that mimics Cds1 checkpoint phosphorylation and constitutively activates MBF [5]. For a strain that lacks fork stabilization function but retains cell-cycle arrest and constitutive G1/S transcription, we used *cds1*Δ *cdc10-2E*. Finally, for a strain in which only cell-cycle arrest is compromised, we use an *nmt1:pyp3 mik1*Δ. In this strain, regulation of the two major checkpoint targets required for preventing mitosis, Cdc25 and Mik1, is disrupted [12]. Regulation of Cdc25, the tyrosine phosphatase responsible for dephosphorylating Cdc2 and driving entry into mitosis, is disrupted by constitutive expression of Pyp3, another phosphatase that can dephosphorylate Cdc2 even if Cdc25 is inhibited by the checkpoint. Regulation of Mik1, a Cdc2 tyrosine kinase activated by the checkpoint, is disrupted by deletion. Therefore *nmt1:pyp3 mik1*Δ cells do not arrest in G2 upon activation of the checkpoints.

**Table 2:** S-phase checkpoint separation-of-function strains.

	Transcription	G2 Arrest	Fork Stability
wild type	+	+	+
*nmt1:pyp3 mik1*Δ	+	−	+
*cds1*Δ *cdc10-2E*	+	+	−
*cds1*Δ	−	+	−
*rad3*Δ *cdc10-2E*	+	−	−
*rad3*Δ	−	−	−

**Figure 2:**
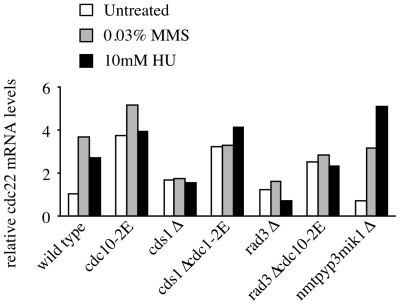
*cdc22* mRNA transcription is up-regulated in a checkpoint- and MBF-dependent manner. Northern blot analysis of *cdc22* transcript levels in asynchronous wild-type (yFS625), *cdc10-2E* (yFS626), *cds1*Δ (yFS627), *cds1*Δ *cdc10-2E* (yFS629), *rad3*Δ (yFS628), *rad3*Δ *cdc10-2E* (yFS630) and *nmt1:pyp3 mik1*Δ (yFS632) cells untreated or treated with 10 mM HU or 0.03% MMS for 4 hours.

In order to confirm that introduction of the *cdc10-2E* allele confers constitutive activation of MBF-dependent gene transcription in our strains, we measured the expression of *cdc22* in asynchronous cultures. Samples were collected from asynchronously growing culture treated with 10 mM HU or 0.03% MMS for 4 hours. As previously reported, wild type cells up-regulate *cdc22* transcription about 4 fold upon checkpoint activation [5]. Maintenance of S-phase transcription is checkpoint dependent, as both *rad3*Δ and *cds1*Δ are unable to maintain high transcription of *cdc22* (Figure 2). Conversely all of the *cdc10-2E* strains constitutively express *cdc22* mRNA. These data confirm that MBF-dependent transcription is regulated by both the replication checkpoint and the S-phase DNA damage checkpoint.

**Figure 3:**
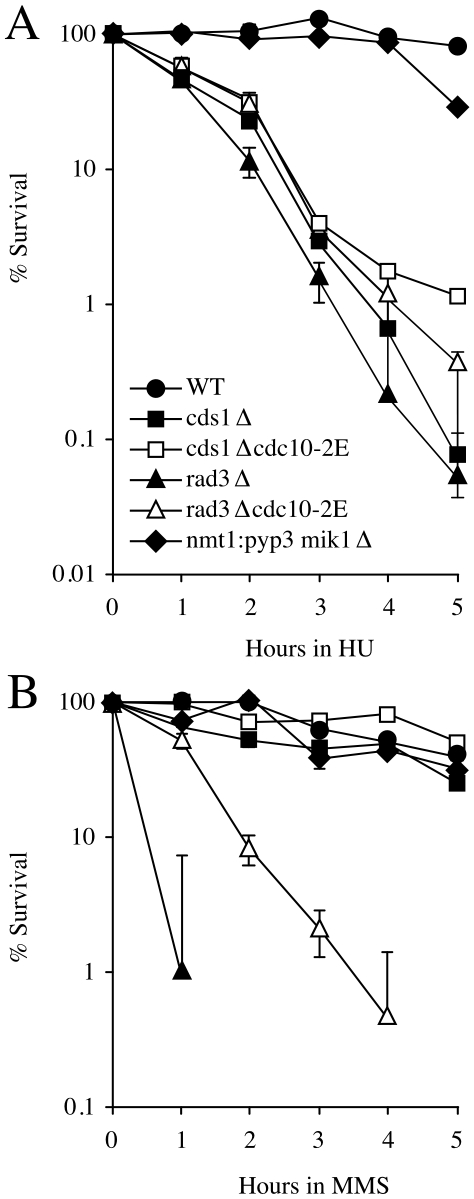
Importance of the transcriptional function of the S-phase checkpoints. Wild-type (yFS625), *cds1*Δ (yFS627), *cds1*Δ *cdc10-2E* (yFS629), *rad3*Δ (yFS628), *rad3*Δ *cdc10-2E* (yFS630) and *nmt1:pyp3 mik1*Δ (yFS632) cells were treated with 10 mM HU or 0.03% MMS for the indicated times and then plated. Colonies were counted after 7 days. The relative percentage survival was calculated by dividing the number of viable colonies from each timepoint by the number of viable colonies before the addition of HU or MMS. n = 3; error bars indicate SEM and are often obscured by the data symbols.

To test the effect of MBF-dependent transcription on viability during replicative stress, we compared the survival of *rad3*Δ or *cds1*Δ cells with that of *rad3*Δ *cdc10-2E* or *cds1*Δ *cdc10-2E* in HU and MMS. *rad3*Δ and *cds1*Δ cells are extremely sensitive to HU, with *cds1*Δ cells being slightly but reproducibly less sensitive (Figure 3A). In both strains, the constitutive expression of G1/S transcripts caused by *cdc10-2E* was modestly but reproducibly beneficial for survival (Figure 3A). *rad3*Δ cells are also extremely sensitive to MMS and constitutive expression of G1/S transcripts makes these cell moderately more resistant (Figure 3B). In contrast, *cds1*Δ cells are quite resistant to MMS. Nonetheless, constitutive expression of G1/S transcripts also makes these cell moderately more resistant (Figure 3B).

To test the importance of cell-cycle arrest in the S-phase checkpoints, we measured the sensitivity of *nmt1:pyp3 mik1*Δ cells to HU and MMS. *nmt1:pyp3 mik1*Δ cells are not acutely sensitive to either treatment. They lose viability only after 5 hours in HU, as the cells accumulate with a cut phenotype caused by cytokinesis after an abnormal mitosis of the unreplicated genome. This result is consistent with the observation that *rad3*Δ cells, which lack the cell-cycle arrest, are not dramatically more sensitive to HU than *cds1*Δ cells, which retain it (Figure 3A). Furthermore, *nmt1:pyp3 mik1*Δ are not significantly more sensitive to MMS at any point in the time course (Figure 3B).

### The MBF-Dependent Transcripts *mik1* and *mrc1* are Involved in Resistance to Replicative Stress

To test the importance of specific G1/S transcripts, we repeated the sensitivity experiments in strains lacking either of two MBF-dependent transcripts: *mik1*, which encodes a kinase that inhibits the G2/M transition by phosphorylating Cdc2, and *mrc1*, a protein involved in Cds1 activation and fork stabilization. We chose these two genes because the S-phase expression of both have been proposed to play checkpoint-independent roles in resistance to replicative stress, suggesting that their continued checkpoint-dependent expression may be a critical role of the transcriptional function of the S-phase checkpoints [13], [14].

**Figure 4:**
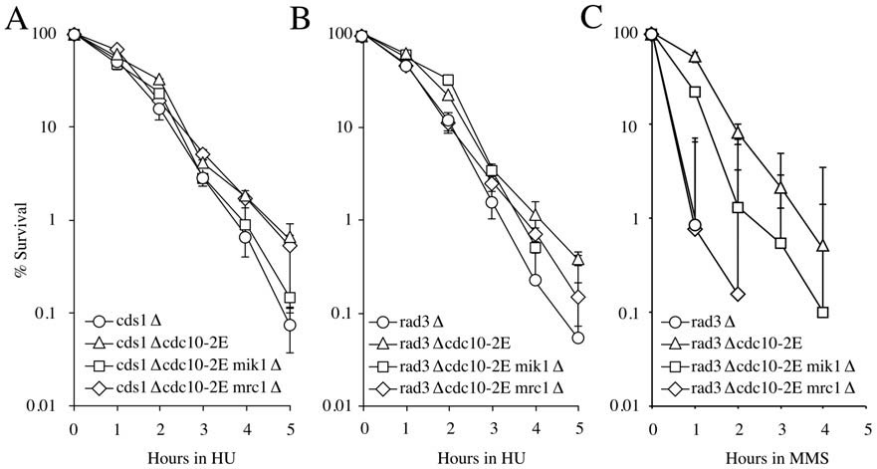
Importance of *mik1* and *mrc1* in the transcriptional function of the S-phase checkpoints. *cds1*Δ (yFS627), *cds1*Δ cdc10-2E</emph> (yFS629), *cds1Δ cdc10-2E mik1Δ (yFS644), cds1Δ cdc10-2E mrc1 (yFS646), rad3Δ (yFS628), rad3Δ cdc10-2E (yFS630), rad3Δ cdc10-2E mik1Δ (yFS643) and rad3Δ cdc10-2E mrc1Δ (yFS645) cells were treated with 10 mM HU or 0.03% MMS for the indicated times and analyzed as in Figure 3.*

Expression of *mik1* plays a role in resistance to both HU and MMS. In the *cds1*Δ background, which retains a Chk1-dependent cell-cycle arrest that can regulate Mik1 [11], [15], constitutive expression of *mik1* plays an important role. It is responsible for the bulk of the *cdc10-2E*-dependent resistance to HU (Figure 4A). In the *rad3*Δ background, which lacks any cell-cycle arrest, constitutive expression of *mik1* provides modest resistance to both treatments, accounting for about half of the resistance due to *cdc10-2E*-dependent G1/S transcript expression (Figure 4B,C).

The expression of *mrc1* also plays a role in resistance to both HU and MMS. In the *rad3*Δ background, *mrc1* expression provides modest resistance to HU, and it is required for all of the *cdc10-2E*-dependent resistance to MMS (Figure 4A,C). However, in the *cds1*Δ background, it plays no significant role in resistance to HU.

### The MBF Target *mik1* Delays Premature Mitosis

**Figure 5:**
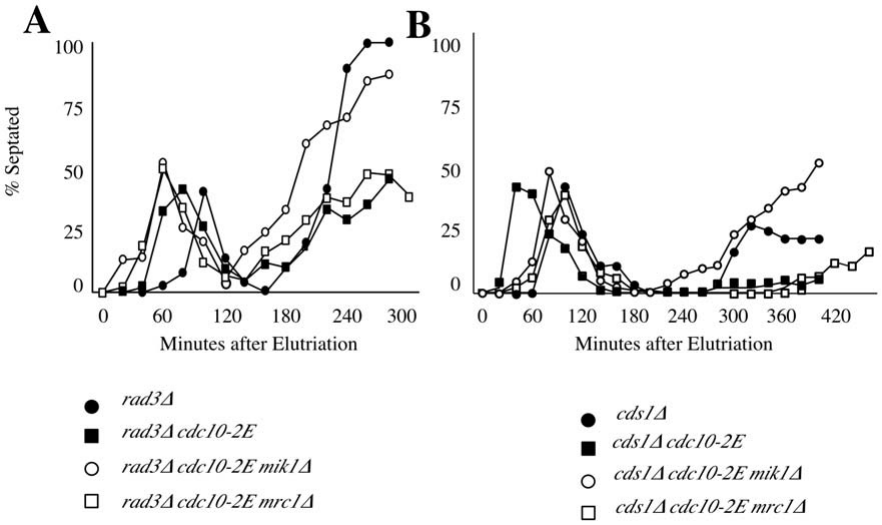
*mik1* plays major role in reducing premature mitosis in *rad3*Δ *cdc10-2E* and *cds1*Δ *cdc10-2E* cells. *cds1*Δ (yFS627), *cds1*Δ *cdc10-2E* (yFS629), *cds1*Δ *cdc10-2E mik1*Δ (yFS644), *cds1*Δ *cdc10-2E mrc1* (yFS646), *rad3*Δ (yFS628), *rad3*Δ *cdc10-2E* (yFS630), *rad3*Δ *cdc10-2E mik1*Δ (yFS643) and *rad3*Δ *cdc10-2E mrc1*Δ (yFS645) cells were synchronized in early G2 by centrifugal elutriation and followed through a synchronous cell cycle in the presence or absence of 10 mM HU. Samples were taken every 20 minutes for visual inspection of septation.

To better understand the role of Mik1 in resistance to replicative stress, we examined the cell-cycle phenotypes of our strains. We synchronized cells in G2, treated them with 10 mM HU and followed cell-cycle progression by septation index for two consecutive cell cycles (Figure 5). As previously reported, *rad3*Δ cells arrested in S phase with HU enter a premature mitosis, producing a so-called cut phenotype, between 180 and 240 minutes (Figure 5A)[16]. The *rad3*Δ *cdc10-2E* double mutant cells begin to cut at approximately the same time, but the percent of cut cells was reduced significantly. This reduction in the frequency of premature mitosis requires expression of *mik1*, since the *rad3*Δ *cdc10-2E mik1*Δ cells cut to the same extent as the *rad3*Δ cells. *mrc1* has no apparent role in regulating entry into mitosis, since *rad3*Δ *cdc10-2E mrc1*Δ cells delay mitosis to the same extent as *rad3*Δ *cdc10-2E* cells.

Similarly, we found that *mik1* expression contributes to cell cycle delay in *cds1*Δ *cdc10-2E* cells. *cds1*Δ cells arrest in HU for about 6 hours and then begin to leak into mitosis (Figure 5B). *cds1*Δ *cdc10-2E* cells remain arrested for the duration of the experiment in a *mik1*-dependent manner. *cds1*Δ *cdc10-2E mik1*Δ cells enter mitosis at the same rate as *cds1*Δ cells, whereas *cds1*Δ *cdc10-2E mrc1*Δ cells enter mitosis at the same rate as *cds1*Δ *cdc10-2E* cells.

### The MBF Target *mrc1* is Involved in Recovery from Replication Stress

**Figure 6:**
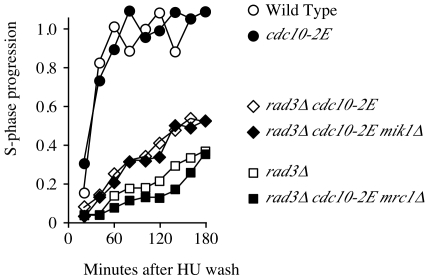
*mrc1* plays major role in recovery from replicative stress in *rad3*Δ *cdc10-2E* cells. *rad3*Δ (yFS628), *rad3*Δ *cdc10-2E* (yFS630), *rad3*Δ *cdc10-2E mrc1*Δ (yFS645) and *rad3*Δ </emph>cdc10-2E mik1</emph>Δ (yFS643) cells were treated with 10 mM HU for 4 hours, washed, resuspended in fresh media and collected every 20 minutes for flow cytometry. S-phase progression was calculated by measuring the mean of the S-phase peak as a percentage of the position of between the means of the 1C and 2C contents.

Mrc1 has two functions: it plays an important role in transmitting checkpoint signaling during S phase and it has an important checkpoint-independent role during replication [14], [17]. To test the hypothesis that constitutive expression of *mrc1* contributes to a checkpoint-independent stabilization of replication forks, we assayed for recovery of replication from an HU arrest (Figure 6). We treated asynchronous cultures with 10 mM HU for 4 hours, released them in fresh media and collected sample for flow cytometry for every 20 minutes. The wild type cells and *cdc10-2E* cells finished replication within 60 minutes after release from HU block. *rad3*Δ cells do not recover well from HU arrest, achieving an average of less than 40% replicated after 180 minutes. *rad3*Δ *cdc10-2E* cells recover better from HU arrest, replicating faster than *rad3*Δ cell and reaching greater than 50% replicated by 180 minutes. To determine the beneficial role of *mik1* and *mrc1* in *rad3*Δ *cdc10-2E* cells, we performed the same experiment with *rad3*Δ *cdc10-2E mrc1*Δ and *rad3*Δ *cdc10-2E mik1*Δ cells. We observed S-phase progression in *rad3*Δ *cdc10-2E mik1*Δ cells similar to *rad3*Δ *cdc10-2E*. However S-phase progression in *rad3*Δ *cdc10-2E mrc1*Δ cells is similar to *rad3*Δ alone, suggesting that the increased recovery provided by transcription of MBF target genes is due primarily to the expression of *mrc1*.

## Discussion

In this study, we examine the role of checkpoint regulation of G1/S transcription in the cellular response to replication stress. We show that G1/S transcription is regulated in response to DNA damage, as well as replication inhibition, during S phase, but that this regulation is not a general DNA damage response, since it is not activated in response to DNA damage during G2. We further compare the relative importance of the three major function of the S-phase checkpoint – cell-cycle arrest, fork stabilization and transcriptional regulation – and show that, although cell cycle regulation and transcription contribute to resistance to replication stress, they are minor factors compared to fork stabilization in response to both DNA damage and replication arrest. Finally, we analyze the specific contribution of two G1/S transcripts to the checkpoints and identify specific roles for them in replicative stress resistance.

In response to replicative stress, the S-phase checkpoints maintains S-phase levels of MBF-dependent G1/S transcription. In previous work, we showed that activation of the HU-induced replication checkpoint maintains S-phase levels of MBF-dependent G1/S transcription by directly regulating the MBF transcription factor [5]. In particular, we showed that *cdc10-2E* constitutively activates MBF-dependent G1/S transcription. *cdc10-2E* encodes an allele of the Cdc10 subunit of the MBF G1/S transcription factor that mimics Cds1 checkpoint phosphorylation by the substitution of two serines with glutamates. Here, we show that MBF-dependent transcription is also regulated by the S-phase DNA damage checkpoint. Transcription of *cdc22*, the canonical MBF transcript encoding the large subunit of ribonucleotide reductase, is maintained during MMS-induced activation of the S-phase DNA damage checkpoint (Figure 1). This regulation is checkpoint-dependent and MMS treatment does not further induce *cdc22* expression in *cdc10-2E*, which mimics checkpoint activation of MBF, suggesting that there is no MBF-independent checkpoint regulation of *cdc22* (Figure 2). However, this regulation of MBF is not a general checkpoint response, because *cdc22* is not up regulated by activation of the G2 DNA damage checkpoint. Although 200 gray of ionizing radiation fully activates the G2 DNA damage checkpoint [18] and in our experiments imposes a 40-minute cell-cycle delay, it does not induce MBF-dependent transcription (Figure 1). Previous work showed modest induction of MBF-dependent transcripts 60 minutes after irradiation with 500 grays, suggesting that MBF may be regulated by chronic activation of the G2 DNA checkpoint [19].

To investigate the contribution of checkpoint-regulated transcription to cellular survival of replicative stress relative to the two other major checkpoint function, cell-cycle arrest and fork stabilization, we built strains that lack one or more of these functions (Table 2). *rad3*Δ cells lack all checkpoint functions. *cds1*Δ cells retain the cell-cycle arrest function, because, in the absence of Cds1, Rad3 can arrest cells via Chk1. *nmt1:pyp3 mik1*Δ cells lack the cell-cycle arrest function because they lack both G2 targets of the checkpoints [12]. In addition, it should be noted that this arrest defect affects not only the direct cell-cycle arrest function of the checkpoint, but also the indirect cell-cycle arrest function provided by checkpoint-dependent expression of *mik1* [see below and 20], [21]. G1/S transcription can be restored in *rad3*Δ and *cds1*Δ cells by mimicking checkpoint phosphorylation of MBF with the *cdc10-2E* allele. We have been unable to test a strain that lacks only the transcription function because we have not been able to identify mutations that prevent checkpoint-dependent MBF activation without also compromising normal cell-cycle MBF regulation [5]. Although these strains generally affect the function described above, it should be noted that they may also affect other currently unrecognized functions. Nonetheless, in broad outline they serve to suggest which of the known functions of the checkpoint are most important in dealing with replicative stress.

We tested these separation-of-function strains for their sensitivity to acute exposure to both HU, which causes nucleotide depletion and replication arrests, but does not directly damage DNA, and MMS, which causes alkylation damage to DNA. Both *rad3*Δ and *cds1*Δ cells are extremely sensitive to HU. The small but reproducible difference between the sensitivity to HU of *rad3*Δ cells, which lack all checkpoint function, and *cds1*Δ cells, which lack the fork-stabilization and transcriptional functions, but retain the cell-cycle arrest function, suggest that cell-cycle arrest provides a modest resistance to HU, presumably by delaying premature mitosis in some cells long enough for them to survive until they are returned to HU-free media (Figure 3). This result is consistent with the observation that *nmt1:pyp3 mik1* cells, which lack only the cell-cycle arrest function, are resistant to brief exposure to HU, but are more sensitive at later timepoints, with 72% lethality at 5 hours, and are sensitive to chronic exposure to HU (CD, unpublished data). Presumably, during brief HU exposure, fork stabilization is sufficient for survival, allowing cells to restart replication once the HU is removed, but at later timepoints premature mitosis kills cells even with stably stalled forks [22].

Restoration of extended MBF-dependent transcription provides modest, but reproducible, resistance to HU in both *rad3*Δ and *cds1*Δ cells, comparable in degree to the resistance provided by the cell-cycle arrest function of the checkpoint. Comparisons between *cds1*Δ and *cds1*Δ *cdc10-2E*, *rad3*Δ and *rad3*Δ *cdc10-2E*, *rad3*Δ and *cds1*Δ, and *nmt1:pyp3 mik1* and wild-type cells all show a roughly 3- to 10-fold increase in sensitivity when the cell-cycle or transcriptional function is removed (Figure 3A). These are quite modest effects with uncertain biological significance. However, they do agree with previous work showing a role for transcriptional regulation in resistance to chronic HU treatment [4], [5]. Furthermore, over-expression of Res2, an MBF subunit and MBF target, suppresses the sensitivity of checkpoint mutants to chronic HU exposure, possibly by constitutively activating MBF-dependent transcription [6]. In contrast to the modest effect of transcriptional regulation, loss of the fork-stabilization phenotype is associated with a increased sensitivity of several orders of magnitude (Figure 3A). These conclusions are consistent with previous work showing recovery from replicative stress is a critical function of the checkpoint [22], [23].

In response to MMS-induced S-phase DNA damage, cell-cycle arrest and fork stabilization appear to play redundant roles in providing resistance. Loss of neither cell-cycle arrest in *nmt1:pyp3 mik1* cells nor fork stabilization in *cds1*Δ cells causes significant sensitivity to MMS (Figure 3B). However, loss of both functions in *rad3*Δ cells leads to profound sensitivity. These results suggest that cells can survive MMS-induced DNA damage either by stabilizing forks and repairing the damage in S phase or by arresting in G2 and repairing the damage caused by lack of fork stabilization. Furthermore, transcription plays a more significant role in response to MMS than HU, suggesting that one or more MBF-dependent transcript can provide checkpoint-independent resistance to MMS-induced DNA damage.

The role of checkpoint regulation of S-phase transcription in resistance to replicative stress raises the question of which specific transcripts provide this resistance. It is unlikely that all transcripts are beneficial. For instance, continued expression of the *cdc18* replication-initiation gene might be expected to complicate recovery from replication stress by promoting refiring of origins [24]. On the other hand the genes encoding Mik1 and Mrc1 are strong candidate for playing an important role in resistance to replication stress [13], [14]. To investigate the role of specific MBF-dependent transcripts in resistance to replicative stress, we repeated the acute sensitivity assays in strains lacking either *mik1*, which encodes a tyrosine kinase that phosphorylates Cdc2 and inhibits mitosis [25], or *mrc1*, which encodes a replication fork protein involved in fork stabilization [14], [17]. We chose these two genes because the S-phase expression of both have been proposed to play checkpoint-independent roles in resistance to replicative stress, suggesting that their continued checkpoint-dependent expression may be a critical role of the transcriptional function of the S-phase checkpoints [13], [14]. Our approach of deleting the genes in question suffers from the complication that we lose both basal and induced expression, but it does test whether, in the absence of these genes, checkpoint-dependent expression of other genes can contribute to replicative-stress resistance. Nonetheless, in future work it would be interesting to express these genes from appropriately-strong constitutive promoters to directly test the importance of their induction.

The checkpoint-dependent transcription of both *mik1* and *mrc1* provide resistance to replicative stress. In the *cds1*Δ background in response to HU, the effect is due primarily to *mik1*, with *cds1*Δ *cdc10-2E mrc1*Δ cells being as resistant as *cds1*Δ *cdc10-2E* cells, but *cds1*Δ *cdc10-2E mik1*Δ cells being as sensitive as *cds1*Δ cells. These results are consistent with the *cds1*Δ cells being able to regulate Mik1 in a Chk1-dependent manner, but only if it is expressed [15].

The checkpoint-dependent transcription of *mik1* also provides resistance to replicative stress in the *rad3*Δ background, in which other cell-cycle arrest mechanisms are abrogated. In response to both HU and MMS, *rad3*Δ *cdc10-2E mik1*Δ cells are more sensitive than *rad3*Δ *cdc10-2E* cells. The role of Mik1 in providing resistance to replicative stress in the absence of the checkpoint is presumably to prevent premature mitosis by inhibiting Cdc2 via tyrosine phosphorylation (Figure 5 and[13]). This conclusion is consistent with the observation that constitutively expressed Mik1 kinase in *cdc10*Δ*C4* (a dominant allele of cdc10 resulting in constitutive over expression of MBF-regulated genes) plays a role in increased cell length in division [21].

The importance of *mrc1* as a transcriptional target of the checkpoints is apparent in the *rad3*Δ background. In response to MMS, *rad3*Δ *cdc10-2E mrc1*Δ cells are as sensitive as *rad3*Δ cells. The situation is somewhat different in the *rad3*Δ background in response to HU. Here, the *rad3*Δ *cdc10-2E mrc1*Δ cells are slightly less sensitive than *rad3*Δ cells, suggesting that other MBF-dependent transcript contribute to resistance in the absence of *mrc1*. However, the *rad3*Δ *cdc10-2E mrc1*Δ cells are as sensitive to HU as *cds1*Δ cells, suggesting that loss of *mrc1* inactivates the Cds1-dependent fork-stabilization function and that the MBF-dependent resistance to HU is due to another function, possibly the cell-cycle arrest function. The fact that *mrc1* expression effects *rad3*Δ cells, but not *cds1*Δ cell, suggests that Mrc1 requires Cds1 in a checkpoint-independent manner for at least some of its fork stabilization functions. The role of Mrc1 in providing resistance to replicative stress in the absence of the checkpoint is presumably to stabilize stalled forks and allow them to recover (Figure 6 and [26]). In particular, Mrc1 may negatively regulate Cdc45 and the MCM helicase, rendering stalled forks capable of resuming replication [27].

Our results show that the regulation of MBF-dependent G1/S transcription by the S-phase checkpoints provides a modest but reproducible resistance to replicative stress. Furthermore, they implicate *mrc1* and *mik1* as critical targets of MBF in the transcriptional response to replication stress. Since both Mrc1 and Mik1, as well as the checkpoint regulation of G1/S transcription, are conserved throughout eukaryotes, it is likely that this mechanism of resistance is a general response to replicative stress.
